# Positive Psychology for Mental Wellbeing of UK Therapeutic Students: Relationships with Engagement, Motivation, Resilience and Self-Compassion

**DOI:** 10.1007/s11469-020-00466-y

**Published:** 2021-01-12

**Authors:** Yasuhiro Kotera, Pauline Green, David Sheffield

**Affiliations:** Human Sciences Research Centre, University of Derby, Kedleston Road, Derby, DE22 1GB UK

**Keywords:** Mental wellbeing, Positive psychology, Resilience, Self-compassion, Intrinsic motivation

## Abstract

This study aimed to examine the relationships between mental wellbeing and positive psychological constructs in therapeutic students (psychotherapy and occupational therapy students). The number of therapeutic students has increased recently; however, they suffer from poor mental health, which may be improved by potentiating their positive psychological constructs, bypassing mental health shame. Therapeutic students (*n* = 145) completed measures regarding positive psychological constructs, namely mental wellbeing, engagement, motivation, resilience, and self-compassion. Resilience and self-compassion predicted mental wellbeing, explaining a large effect. Self-compassion partially mediated the relationship between resilience and mental wellbeing. This study highlights the importance of positive psychological constructs, especially resilience and self-compassion, for mental wellbeing of therapeutic students.

In the UK, caring profession subjects—related to humans taking care of other humans, whether physically, mentally or spiritually (Kotera et al. 2018d)—are the most popular of all university disciplines. Of 700,000 students who applied to undergraduate programmes in UK universities, 60% of them applied to this discipline in 2014 (McGhee 2015). More than one in every five students studied either an undergraduate or postgraduate caring profession subject programme in the academic year 2015–2016 (Higher Education Statistics Agency 2017). This is unique to the UK. For example, business is the most popular subject in the USA (National Center for Education Statistics 2016) and in Germany (Muller 2016) and social science is most popular in Japan (Ministry of Education, Culture, Sports, Science and Technology-Japan 2015). One notable reason for this popularity in caring subjects in the UK relates to stable post-study employment: for instance, almost all the occupational therapy students are employed within 6 months of graduation (Association of Graduate Careers Advisory Services 2017).

Caring profession subjects include allied health (e.g. occupational therapy, physiotherapy), psychotherapy, nursing, social work and teaching (Kotera et al. 2018d). Despite its popularity, the mental wellbeing of caring subject students is challenging: UK social work students have high levels of depression, anxiety and stress (Kotera et al. 2019b); nursing students have poor mental wellbeing (Kotera et al. 2019); and more than one-third of occupational therapy and psychotherapy students have depression and low self-esteem (Boellinghaus et al. 2013). As these students are dealing, or will deal with patients’ lives, high rates of poor mental wellbeing, which can cause poor decisions (Blackmore et al. 2007; Brooks et al. 2002) is alarming. Mentally distressed healthcare workers are more likely to make errors, negatively impacting on client safety, which is the central element in education for caring profession (Melnyk et al. 2018; The Quality Assurance Agency for Higher Education 2013). Moreover, this relates to the current emphasis, associated with coronavirus disease 2019 (COVID-19), that caring professionals need to take care of themselves in order to offer care for others (Kotera et al. 2020a, b; Spoorthy et al. 2020).

Among the caring disciplines, this study focused on therapeutic students (psychotherapy students and occupational therapy students; Kotera et al. 2019c), because the number of students in these programmes have expanded recently due to the government’s prioritised focus on mental health (Complete University Guide 2018).

## Usefulness of Positive Psychology for Mental Wellbeing

One contributing factor to caring students’ poor mental wellbeing may be their hesitancy to ask for help. For example, high caregiver identity was related to high shame about mental health problems (mental health shame; Kotera et al. 2019d) among students in caring subjects (Kotera et al. 2019b). Further, mental health shame was a positive predictor of mental health problems in therapeutic students (Kotera et al. 2019c). This may imply that offering these students ‘mental health’ support directly would not be effective, as they would not fully engage in such support because of shame (Kotera et al. 2018b). On the other hand, positive psychological approaches may be effective as these approaches could bypass their high mental health shame, focusing on nurturing what assets they have (Joseph and Linley 2006; Kotera and Ting 2019). Indeed, the importance of positive psychology has been recognised among therapeutic practitioners (Bannigan 2002; Joseph and Linley 2006; Maki and Endo 2018).

Positive psychology focuses on happiness and positivity, aiming to further strengthen one’s strengths and values (Seligman and Csikszentmihalyi 2000), as opposed to traditional psychology which primarily focuses on pathologies (i.e. what is impaired), aiming to dissect them out. Positive psychology perceives that all people have the potential (given the right skills and contexts), instead of focusing on what people lack (Kashdan and Ciarrochi 2013). Positive psychological approaches are recommended to prevent mental health problems in the general population (Forsman et al. 2015; Kobau et al. 2011) and in UK students (Denovan and Macaskill 2017).

Intervention studies have reported promising results. Acceptance and commitment therapy increased positive psychological outcomes and decreased depression in a randomised controlled trial (Bohlmeijer et al. 2015). A 5-week online positive psychology intervention, focusing on positive emotions (e.g. engagement and meaning), increased pregnant women’s life satisfaction and reduced depression (Corno et al. 2018). PhD students’ mental distress was alleviated after 8-week engagement and motivation training (Marais et al. 2018). Moreover, a 10-year longitudinal study on adults’ positive psychology and mental distress noted a relationship between heightened positive psychology constructs and mitigated mental distress (Keyes et al. 2010). Likewise, positive psychological constructs predicted large variance (28–53%) of mood disorders in a 3-year longitudinal study (Schotanus-Dijkstra et al. 2016). It is unsurprising that positive psychology has been recommended to counsellors who help empower clients to accomplish mental health, wellbeing and career goals (American Psychological Association 2015) by increasing their happiness and developing their strengths (Haktanir et al. 2016). These findings support the significant relationships between positive psychology constructs and mental wellbeing.

Academic engagement (hereafter ‘engagement’) is a particularly important positive psychological construct in higher education for its associations with various student outcomes including mental health (Liébana-Presa et al. 2014; Rogers et al. 2017), attainment (Casuso-Holgado et al. 2013; Neel and Fuligni 2013) and intrinsic motivation (Armbruster et al. 2009; Bicket et al. 2010). Engagement refers to the degree students are committed to make an effort in their academic work (Newman et al. 1992), and is related to mental health in 410 Australian students (Turner et al. 2017). Students’ commitment to their academic work is positively related to their mental wellbeing. The positive associations between engagement and mental wellbeing have been found in student populations in other countries too (e.g., Datu 2018; Suárez-Colorado et al. 2019). However, research into these relationships in UK university students has been scarce, particularly in UK therapeutic students.

Intrinsic motivation, closely related with engagement, is another key positive psychological construct, related to mental wellbeing (Baard et al. 2004; Bailey and Phillips 2016; Locke and Latham 2004). Intrinsic motivation, opposing extrinsic motivation, is one type of motivation in Self-Determination Theory, one of the most researched motivation theories (Deci and Ryan 1985). Self-Determination Theory presumes that every individual has an innate predisposition to direct their psychological power to self-actualisation, while maintaining social connections (Deci and Ryan 1985). Intrinsic motivation is present in activities that are naturally satisfying and fulfilling (i.e., being involved in the activity itself is a reward), whereas extrinsic motivation is relevant to activities that are a means to an end (e.g., money and status that can be brought as a result of the activities; Kotera et al. 2018d).

Intrinsic motivation is related to positive outcomes including better performance (Baard et al. 2004), wellbeing (Bailey and Phillips 2016), life satisfaction (Locke and Latham 2004), prosocial behaviour (Gagné 2003) and ethical judgement (Kotera et al. 2018a). In contrast, extrinsic motivation is related to negative outcomes such as burnout (Houkes et al. 2003), shame (Kotera et al. 2018a), depression (Blais et al. 1993), compromised performance (Vallerand 1997) and poor ethical judgement (Kotera et al. 2018a). Students’ intrinsic motivation was related to academic performance in Israeli nursing students (Khalaila 2015) and meaningfulness in Norwegian health and social work students (Utvær 2014); however, these relationships have not been thoroughly investigated in UK therapeutic students in relation to mental health.

Though emotional resilience (hereafter ‘resilience’) has been defined in varied ways (e.g. Pooley and Cohen 2010; Ungar 2008), resilience is considered a generic term entailing internal resources to help an individual to overcome adversity, and expand themselves from such experiences (Grant and Kinman 2014). The importance of resilience in practice has been noted in the professional ethical and capability framework (e.g. British Association for Counselling and Psychotherapy 2018; College of Occupational Therapists 2014). Resilient practitioners are aware of their strengths and agency, and are able to reframe adversity as an opportunity for growth (Russ et al. 2009; Harrison 2013). Indeed, resilient people are not exempt from being affected by life challenges; however, they tend not to be overwhelmed by those challenges permanently (Tugade and Fredrickson 2004). Enhancing resilience was associated with better mental wellbeing, along with improved psychological outcomes such as self-efficacy, mindfulness and compassion (Robertson et al. 2015). Resilience can be maintained with psychological resources such as motivation, reframing life challenges into growing experiences (Bryan et al. 2017)—essential to therapeutic students (British Association for Counselling and Psychotherapy 2018; College of Occupational Therapists 2014).

Lastly, self-compassion has been increasingly paid attention to, for its relevance to mental wellbeing (Ehret et al. 2015; Kotera et al. 2018b, 2019; Muris et al. 2018). Self-compassion—being understanding and accepting of one’s weaknesses and inadequacies (Gilbert 2009)—protects mental wellbeing by supporting resilience (Trompetter et al. 2017). Self-compassion is strongly related to mental wellbeing (Ehret et al. 2015; Hayter and Dorstyn 2014; Muris et al. 2018), and a significant predictor of mental wellbeing in UK social work students (Kotera et al. 2019b). In addition, numerous studies reported that self-compassion mediated the relationship between positive mental constructs and mental wellbeing (Beaumont et al. 2016; Fong and Loi 2016; Neff and Mcgehee 2010; Zhang et al. 2016), suggesting that self-compassion would be a mediator of the relationship between resilience and mental wellbeing (Trompetter et al. 2017). Resilience Theory posits that how we cope with difficulties, rather than the difficulties themselves, matters more to our wellbeing (Van Breda 2018). Treating oneself in a caring manner is conducive to one’s coping, emphasising one’s strengths in the process of resilience (Fergus and Zimmerman 2005). Moreover, Social Mentality Theory suggests how we treat ourselves originates from how we treat others (Gilbert 2000). Therapeutic students aspire to help others; therefore, based on this theory, they may be also willing to help themselves. Accordingly, whether self-compassion would mediate the relationship between resilience and mental wellbeing in therapeutic students needs to be examined.

Despite the significant relationships between mental wellbeing and these positive psychological constructs, these have not yet been explored among UK therapeutic students in depth. This study, therefore, aimed to explore the relationships between mental wellbeing, engagement, motivation, resilience and self-compassion. Two sets of hypotheses were tested:

H1: Mental wellbeing will be associated with (a) engagement, (b) motivation, (c) resilience and (d) self-compassion.

H2: Self-compassion will mediate the relationship between resilience and mental wellbeing.

## Methods

### Participants

Participants were aged 18 years or older and enrolled on an occupational therapy or psychotherapy programme at a UK university in the Midlands region. One hundred and forty-five students of 166 full-time students completed the self-report measures about wellbeing, engagement, motivation, resilience and self-compassion. This is a parallel study with another study exploring their mental health attitudes. The findings are reported elsewhere.

### Materials

Mental wellbeing was evaluated using the seven-item *Short Warwick*–*Edinburgh Mental Wellbeing Scale* (*SWEMWBS*; Stewart-Brown et al. 2009), shortened version of the original 14-item scale (Stewart-Brown and Janmohamed 2008). Though how to measure mental wellbeing is still open to debate, this scale is one of the most established wellbeing scales, and its holistic appraisal on mental wellbeing fits with the purpose of this present study (Cooke et al. 2016). Reflecting on the past 2 weeks, students respond to the seven items, regarding both hedonic and eudaimonic wellbeing (e.g., ‘I’ve been dealing with problems well’), on a five-point Likert scale (1 = ‘None of the time’ to 5 = ‘All of the time’). SWEMWBS had high internal consistency (α = .85; Stewart-Brown et al. 2009).

Engagement was examined using the *Utrecht Work Engagement Scale for Students* (*UWES-S*), a 17-item on a seven-point Likert scale (0 = ‘Never’ to 6 = ‘Always (everyday)’) considering how active and confident students feel towards their academic activities (Schaufeli and Bakker 2004). The three subscales of UWES-S relates to vigour, that is mental energy leading to a substantial effort in academic work (six items including ‘I am very resilient, mentally, as far as my studies are concerned’), dedication, namely commitment to academic work (five items including ‘My study inspires me’), and absorption, regarded as positive immersion in academic work (six items including ‘When I am studying, I forget everything else around me.’) (Schaufeli et al. 2002). UWES-S had high internal consistency (α = .63–.81; Schaufeli and Bakker 2004). In this study, the average of the total engagement score was used (Schaufeli and Bakker 2004).

*The Academic Motivation Scale* (*AMS*; Vallerand et al. 1992), comprising 28 items assessing three types of motivation categorised into seven subtypes: (i) amotivation, (ii) extrinsic motivation (external, introjected, and identified regulation) and (iii) intrinsic motivation (to know, to accomplish and to experience stimulation). Students respond to items asking why they go to university (e.g. ‘I don’t know; I can’t understand what I am doing in school’ for amotivation, ‘Because I want to have “the good life” later on.’ for extrinsic motivation, and ‘Because I experience pleasure and satisfaction while learning new things’ for intrinsic motivation; the wording was adjusted to the UK higher education, e.g. ‘school’ was changed to ‘university’), on a seven-point Likert scale (1 = ‘Does not correspond at all’ to 7 = ‘Corresponds exactly’). AMS had adequate to high internal consistency (α = .62–.91; Vallerand et al. 1992).

Resilience was measured using the *Brief Resilience Scale* (*BRS*), a six-item scale measuring the ability to bounce back from difficulties (Smith et al. 2008). Students respond to the six items (e.g. ‘I usually come through difficult times with little trouble’) on five-point Likert scale (1 = ‘Strongly Disagree’ to 5 = ‘Strongly Agree’) for the items 1, 3 and 5, and the rest of the inverted Likert scale (i.e. 5 = ‘Strongly Disagree’ to 1 = ‘Strongly Agree’). BRS had high internal consistency (α = .80–.91; Smith et al. 2008).

Lastly, self-compassion was assessed using the *Self*-*Compassion Scale*-*Short Form* (*SCS*-*SF*; Raes et al. 2011), a 12-item on five-point Likert scale to evaluate how consistently you behave kindly towards yourself in difficult situations (0 = ‘Almost never’ to 5 = ‘Almost always’). Items include ‘I try to be understanding and patient towards those aspects of my personality I don’t like’, and items 1, 4, 8, 9, 11 and 12 are responded inversely (0 = ‘Almost always’ to 5 = ‘Almost never’). SCS-SF had high internal consistency (α = .86; Raes et al. 2011).

### Procedure

Once the consent form had been completed, students were presented with the scales, followed by the debrief. In case students were distressed by attending the study, information about available mental health support, inside and outside the university, was provided, to treat any issues in a careful manner. All materials were provided in hard copy. Ethics approval was obtained from the university research ethics committee.

After screening for the outliers and assumptions of parametric tests, correlations and multiple regression analyses were performed to explore relationships among those constructs. Followingly, path analyses were conducted to identify whether self-compassion mediates the relationship between resilience and mental wellbeing. SPSS version 25 and Process Macro version 3 (Hayes 2018) were used for these analyses.

## Results

Demographic data of our sample were *M*_age_ = 26.80, SD_age_ = 8.64, RNG_age_ = 17–52 years old; 15 males and 130 females; 133 undergraduates and 12 postgraduates; 76 occupational therapy students and 69 psychotherapy students; 131 British, 7 other European, 4 Asian, 2 African and 1 North American. Gender balance of our sample (10% male) was similar to occupational therapists and counsellors (Brown 2017; Grant et al. 2004), maintaining good representativeness.

### Correlations Between Mental Wellbeing, Engagement, Motivation, Resilience and Self-Compassion (H1)

Eight scores in amotivation were identified as outliers, using the outlier labelling rule (Hoaglin and Iglewicz 2012), so were winsorised (Tukey 1962). All variables had high internal consistency (α = .82–.91; Table 1). Since engagement, motivation and self-compassion were not normally distributed (Shapiro-Wilk, *p* < .05), all the sub/scales were square-root-transformed. Pearson’s correlation was calculated to explore the relationships between mental wellbeing, engagement, motivation, resilience and self-compassion (Table 1).

**Table 1:** Descriptive statistics and correlations between mental wellbeing, academic engagement, motivation, resilience and self-compassion in 145 UK therapeutic students

		*M*	SD	α	1	2	3	4	5	6	7	8	9
1	GN (M = 1, F = 2) 15 males, 130 females				–								
2	Age	26.80	8.64		.06	–							
3	Mental wellbeing	23.94	4.85	.85	− .18*	.18*	–						
4	Engagement	3.81	.97	.91	.05	.20*	.32**	–					
5	Intrinsic motivation	18.50	4.64	.82	− .02	.22**	.24**	.52**	–				
6	Extrinsic motivation	20.81	4.23	.87	.02	− .03	.05	.28**	.40**	–			
7	Amotivation	6.08	2.97	.83	− .04	− .01	− .21*	− .33**	− .11	− .06	–		
8	Resilience	19.86	5.58	.83	− .14	.20*	.50**	.27**	.09	− .08	− .13	–	
9	Self-compassion	34.05	9.52	.84	− .16	.15	.61**	.21**	.10	− .22**	− .26**	.60**	–

*p < .05; **p < .01

Mental wellbeing was positively related with engagement, intrinsic motivation, resilience and self-compassion, and negatively related with gender and amotivation. Only extrinsic motivation was not related with mental wellbeing. Engagement was positively related with intrinsic motivation, extrinsic motivation, resilience and self-compassion, while negatively related with amotivation.

### Positive Psychological Predictors of Mental Wellbeing (H1)

To identify predictive relationships with mental wellbeing, multiple regression analyses were performed. Engagement, intrinsic motivation, amotivation, resilience and self-compassion (significant correlates of mental wellbeing) were entered as predictor variables, and mental wellbeing was entered as an outcome variable. Multicollinearity was not a concern (VIFs < 10). These positive psychological predictor variables accounted for 35% for mental wellbeing, a large effect size. Resilience and self-compassion were significant positive predictors for mental wellbeing. Self-compassion was the strongest predictor of mental health among all the positive psychological constructs. H1 was partially supported (H1c and H1d) (Table 2).

**Table 2:** Multiple regression: engagement, motivation, resilience and self-compassion for mental wellbeing in 145 UK therapeutic students

	Mental wellbeing		
	*B*	SE_B_	β
Step 1			
GN (1 = M, 2 = F)	− .21	.09	− .19*
Age	.01	.01	.19*
Adj. *R*^2^	.06		
Step 2			
GN (1 = M, 2 = F)	− .10	.07	− .09
Age	.002	.004	.04
Engagement	.20	.16	.10
Intrinsic motivation	.10	.07	.11
Amotivation	− .03	.07	− .03
Resilience	.14	.06	.18*
Self-compassion	.32	.06	.44**
Δ Adj. *R*^2^	.35		

*p < .05; **p < .01

### Self-Compassion as a Mediator Between Resilience and Mental Wellbeing (H2)

Finally, to examine whether self-compassion mediated the relationship between resilience and wellbeing, path analyses were conducted using model 4 in the Process macro (parallel mediation model; Hayes 2018).

**Fig. 1:**
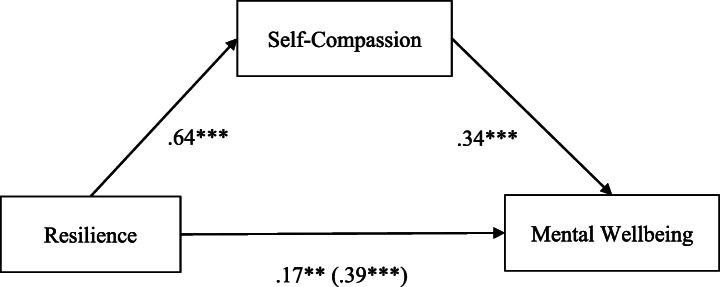
Parallel mediation model: resilience as a predictor of mental wellbeing, mediated by self-compassion. The confidence interval for the indirect effect is a BCa bootstrapped CI based on 5000 samples. **p < .01; ***p < .001; direct effect (total effect)

As summarised in Fig. 1, there was a significant indirect effect of resilience on mental wellbeing through self-compassion, *b* = .22, BCa CI [.13, .33], which explained 15% of the variance in mental wellbeing, and accounted for 56% of the total effect, a large effect size. The direct effect of resilience on mental wellbeing, controlling for self-compassion, was also significant, *b* = .17, *t*(141) = 2.70, *p* = .008, implying that resilience could directly predict the variance in mental wellbeing. The total effect of resilience on mental wellbeing, including self-compassion, was also significant, *b* = .39, *t*(142) = 6.93, *p* < .001. In total, 25% of the variance in mental wellbeing was explained by resilience, controlling for self-compassion. Because all the paths were significant, self-compassion partially mediated the relationship between resilience and mental wellbeing; H2 was supported.

## Discussion

This study investigated the relationships between mental wellbeing and positive psychological constructs, namely engagement, motivation, resilience and self-compassion in UK therapeutic students (i.e. psychotherapy students and occupational therapy students). Their mental wellbeing was positively associated with engagement, intrinsic motivation, resilience and self-compassion, while negatively associated with amotivation. Extrinsic motivation was not associated with mental wellbeing. Self-compassion and resilience were significant predictors of mental wellbeing, and self-compassion was the strongest predictor. Lastly, self-compassion partially mediated the relationship between resilience and mental wellbeing.

### Correlations Between Mental Wellbeing and Positive Psychological Constructs

In line with previous research, all the positive psychological constructs (engagement, intrinsic motivation, resilience and self-compassion) were related to mental wellbeing. Resilience and self-compassion, in particular, had large effect relationships (*r* > .50). Students who are (a) well-engaged with academic work, (b) passionate about academic work, (c) able to bounce back from challenges and (d) understanding towards themselves tend to have a high level of mental wellbeing. Previously, these relationships between mental wellbeing and positive psychology have been reported in other student populations (Kotera and Ting 2019; Kotera et al. 2018b), and our findings report that the relationships were identified in the UK therapeutic students too. These identified relationships can help substantiate practitioners’ experience, noting that positive mental resources are essential in the therapeutic field (Vandenberghe and Silvestre 2014). Both occupational therapists and psychotherapists work in collaboration with other professionals, and a wide range of clients. Considering the contagious nature of positive emotions, recognising positive psychology is useful to trainees and practitioners (Bannigan 2002; Dunn 2017).

This may suggest that educators and university counsellors may need to consider embedding a session or event focusing on potentiating students’ positive psychological resources in their curriculum and services. For example, an 8-week mindfulness training was effective to strengthen resilience to mental distress in a UK general student sample (Galante et al. 2018). Weekly mindfulness sessions over a month improved occupational therapy students’ self-awareness and stress management skills (Stew 2011). These types of training may benefit from considering the holistic nature of positive psychology, reflecting several key areas of student lives (Hall et al. 2015), instead of solely focusing on their academic life. Unsurprisingly, positive psychological approaches were recommended for international therapists to learn as an intervention, for its applicability to diverse client issues (Lenz et al. 2018). Training in positive psychology would be especially beneficial to therapeutic students. Future research needs to explore the effects of such interventions targeting positive psychology, leading to better mental wellbeing.

### Self-Compassion and Resilience Predict Mental Wellbeing

Self-compassion and resilience were identified as predictors of mental wellbeing, and self-compassion was the strongest predictor of all the studied variables. Resilience has been emphasised in the psychotherapy and occupational therapy fields (British Association for Counselling and Psychotherapy 2018; College of Occupational Therapists 2014), and our findings echoed this. Students who study in these programmes are familiar with, and cognisant of the importance of resilience (Kotera et al. 2018a). Resilience research has been active in the recent years; however, little has focused on this student group. The ability to bounce back from the difficulties was deemed to be important to mental wellbeing of the UK therapeutic students. As mentioned above, mindfulness is one way to potentiate resilience (Galante et al. 2018; Stew 2011).

Likewise, self-compassion has been a focus in mental health research recently, and its importance has been reported (Kotera et al. 2018c). In the UK, building a compassionate culture among caring professionals has been endorsed (Department of Health, 2013); however, compassion towards practitioners themselves has been rather under-emphasised (Mckenna and Mellson 2013). Implementing self-compassion training to therapeutic students may be useful, aiming for better self-care and mental wellbeing (Christopher and Maris 2010). For instance, a session during the orientation week may be beneficial and effective, because informing students of mental wellbeing and coping skills in the beginning of their programme could prepare them for the potential academic stress, which can support students’ help-seeking, resulting in better clinical outcomes. Additionally, due to the contagious nature of compassion (Fowler and Christakis 2010), tutors may also benefit from this type of event as compassion was associated with tutors’ mental health (Jennings and Greenberg 2009). Future research should examine the effects of self-compassion training on therapeutic students’ mental health.

### Self-Compassion Mediates Resilience-Mental Wellbeing

Lastly, our path analyses revealed that self-compassion partially mediated the relationship between resilience and mental wellbeing. Self-compassion strengthens the significant impact of resilience on mental wellbeing. Again, this may highlight the importance of these two constructs for mental wellbeing of therapeutic students (Dunn 2017). While resilience is highly recognised in therapeutic students and their professional bodies (British Association for Counselling and Psychotherapy 2018; College of Occupational Therapists 2014), self-compassion is still a new concept (Mckenna and Mellson 2013), indicating a need for more recognition of self-compassion in this field. Future research needs to evaluate the effects of self-compassion education and training on mental wellbeing of therapeutic students.

### Limitations

Several limitations to this study need to be noted. Firstly, the participants were recruited through opportunity sampling at one university, limiting the generalisability of the findings. Secondly, this study examined a combination of occupational therapy and psychotherapy students, as each sample size alone did not reach the required sample size calculated on power analysis (*n* = 114; Faul et al. 2009). Future research should explore these groups of students separately to further understand the specific characteristics of each student group. However, in the current sample, there were no differences in the measures between the groups (*p* ≧ .24). Thirdly, measuring these constructs using self-report measures might compromise their accuracy (e.g. response bias [Kotera et al. 2020a], and validity [Kotera and Sheffield 2020]). Additional qualitative data (e.g. a focus group discussion with students) could offer more in-depth findings. Fourthly, the causal directions of these related psychological constructs have not been evaluated. Longitudinal studies would help understand the temporal patterning of the identified relationships and may help design approaches that would increase our understanding of causality.

## Conclusion

The poor mental wellbeing of this expanding student group may be improved by potentiating their positive psychological constructs, which could bypass their high mental health shame. This study examined the relationships between mental wellbeing and positive psychological constructs, namely academic engagement, motivation, resilience and self-compassion of UK therapeutic students. Their mental wellbeing was associated with engagement, intrinsic motivation, resilience and self-compassion. Self-compassion and resilience were significant predictors of mental wellbeing. Lastly, self-compassion partially mediated the relationship between resilience and mental wellbeing. Findings highlight the importance of positive psychology in a therapeutic student group, and will help educators, practitioners, researchers and students to identify alternative approaches to improve their mental wellbeing.

All procedures followed were in accordance with the ethical standards of the responsible committee on human experimentation (institutional and national) and with the Helsinki Declaration of 1975, as revised in 2000 (5). Informed consent was obtained from all patients for being included in the study.
